# Examining the viability of dorsal fin pigmentation for individual identification of poorly-marked delphinids

**DOI:** 10.1038/s41598-018-30842-7

**Published:** 2018-08-22

**Authors:** M. D. M. Pawley, K. E. Hupman, K. A. Stockin, A. Gilman

**Affiliations:** 1 Institute of Natural and Mathematical Sciences, Massey University, Auckland, 0745 New Zealand; 2 National Institute of Water and Atmospheric Research, 301 Evans Bay Parade, Wellington, 6021 New Zealand

## Abstract

Dolphin photo-identification has traditionally relied only on distinctive markings on the dorsal fin—this is problematic for delphinids whose populations exhibit a low mark ratio. We used common dolphins (genus *Delphinu*s) as a model species to assess the viability of using pigmentation for photo-identification. Using a photo-identification catalogue of 169 adult individuals collected between 2002 and 2013, we extracted features that quantified pigmentation in a manner that was robust to lighting artefacts and dorsal fin orientation. We determined the proportion of individuals which exhibited pigmentation and examined temporal stability by (i) visually examining individuals and (ii) testing for seriation. We found 88–91% of images could be manually matched to the correct individual in the catalogue based on pigmentation patterns alone. A linear discriminant analysis classifier correctly identified the correct individual 77% of the time. We found 95% common dolphins exhibited distinctive pigmentation—all of which were temporarily stable. Our work challenges the current thinking that pigmentation is an unreliable feature for delphinid photo-identification and suggests that this feature could be applied to common dolphins and other poorly-marked delphinids.

## Introduction

The need to monitor cetacean populations for management purposes typically requires assessments of abundance^1,2^, site fidelity^3^, movement patterns^4,5^ and social structure^6^. A range of methods have been used to examine these parameters including: distance-sampling where line-transect surveys are used to count animals to assess their density^7,8^, and mark-recapture (MRC) analysis using sightings (and re-sightings) of naturally or artificially marked individuals as a sample of the population^9^. Distance-sampling techniques assess species at the population level but do not give information about specific individuals. In contrast, MRC methods, including photo-identification (photo-id), samples individuals—which in turn, may give an understanding of individual-level site fidelity, movement patterns and social structure.

Photo-id is a cost-effective approach that utilises unique naturally-occurring marks, eliminating the need to physically capture or tag the organism. This method has been applied to a variety of species through the identification of unique natural features. Historically, the use of photo-id has been highly applied to well-marked coastal delphinids (such as bottlenose dolphins, *Tursiops truncatus*), but less successfully to the poorly-marked, gregarious common dolphins (*Delphinus* spp.) due to the difficulties in identifying individuals.

For most delphinid photo-id studies, the most frequently used identifying feature is nicks and notches present on the leading and trailing edges of the dorsal fin^10,11^. However, the use of nicks and notches for individual identification is problematic for populations which have a low mark ratio (ratio of marked to unmarked dorsal fins). For example, common dolphins are poorly marked and therefore difficult to identify—they have the second lowest mark ratio of any delphinid (ca 10–46% of animals have identifiable dorsal fin nicks/notches^12,13^). This difficulty (along with the general logistical problems associated with studying any large aggregated population) is reflected by the very small number of studies that have been published on *Delphinus* worldwide—only six in total^12,14–18^.

Finding other features unique to individuals could assist in the recognition of poorly-marked delphinids. Increasing the proportion of animals that can be catalogued and providing a secondary feature to confirm matches would likely have a great impact on those studies that rely on individual identification. In this context, we consider pigmentation to be a feature worthy of exploration. Pigmentation has been successfully applied as a feature for individual recognition of delphinids previously (see Table 1), but its use has traditionally been limited, mainly due to three concerns: (1) pigmentation may not be prevalant in populations; (2) pigmentation may be not stable over time, and (3) pigmentation may not have sufficient discriminatory power to identify individuals in the presence of imaging artifacts.

**Table 1:** Studies of delphinids which have used pigmentation patterns for individual identification, specifically outlining if pigmentation prevalence, stability or discriminatory power has been examined via visual or statistical analysis.

Species	Type of study	Identifying feature(s)	Pigmentation prevalance	Pigmenation stability	Discriminatory power of pigmentation	Reference
Baiji dolphin (*Lipotes vexillifer*)	Movement, abundance and threats	Body scratches; dorsal fin nicks and deformities; facial pigment patterns	No	No	No	^28^
Indo-Pacific humpback dolphin (*Sousa chinensis*)	Population differences	Body pigmentation	No	No	No	^31^
Irrawaddy dolphin (*Orcaella brevirostris*)	Photo-identification	Dorsal fin notches, white pigmentation patterns and shapes	Yes	No	No	^29^
Killer whale (*Orcinus orca*)	Photo-identification	Saddle patch	No	No	No	^48^
	Photo-identification	Saddle patch	Yes	Yes (visual)	No	^49^
Pink river dolphin (*Inia geoffrensis*)	Photo-identification	Dorsal fin nicks and notches, pigmentation, wounds, scratches scrapes and bends; body pigmentation, wounds, white or black marks, scratches, scrapes	Yes	Yes (visual)	No	^32^
	Occurrence, habitat and prey	Pigment, marks, notches	No	No	No	^21^
Risso’s dolphin	Photo-identification	Dorsal fin and body marks	No	Yes (visual)	No	^20^
Short-beaked common dolphin (*Delphinus delphis*) & common bottlenose dolphin (*Tursiops truncatus*)	Occurence	Dorsal fin nicks and notches and pigmentation	No	No	No	^15^
Short-beaked common dolphin (*Delphinus delphis*)	Photo-identification	Dorsal fin nicks and notches and pigmentation	No	No	No	^12^
	Occurence	Dorsal fin nicks and notches and pigmentation	No	No	No	^15^
	Abundance	Dorsal fin nicks and notches and pigmentation	No	No	No	^16^
	Abundance, site fidelity, movement, social structure	Dorsal fin nicks and notches and pigmentation	Yes	Yes (visual and statistical analysis)	Yes	^13^
	Residancy	Dorsal fin nicks and notches and pigmentation	No	Yes (visual)	No	^18^
Spotted dolphin (*Stenella frontalis*)	Photo-identification	Body pigmentation	No	No	No	^30^

Some studies have examined pigmentation prevalance within populations and visually assessed pigmentation stability over time (see Table 1 for summary). However, there are no published dolphin studies which have devised methods to quantitatively verify pigmentation stability or measure its discriminatory power when identifying individuals. Moreover, while dorsal fin pigmentation has been used as a secondary feature to confirm individual common dolphins identified based on nicks/notches, its use as a sole feature for identification has never been explored for this species. The main contributions of this work are two-fold: Firstly, we develop an approach to examine the suitability of pigmentation patterns for its use in photo-identification, in particular with computer-assisted methods. This approach quantifiably examines the main concerns that limit the use of dorsal fin pigmentation—namely its prevalence, temporal stability and discriminatory power. Secondly, we apply the developed methodology to the common dolphin to show that this species can be successfully recognised using pigmentation patterns.

## Results

The following results were determined using human observations (manual inspection) of photos:

- prevalence of pigment;
- manual inspection of pigment change;
- discriminatory power of pigmentation - manual matching.

To quantify pigmentation patterns, we used computer vision methods to extract 142 different measurable properties that were representative of the pigmentation patterns (‘features’) found on the dorsal fin images. These features were used to assess the following:

- pigment stability over time (test of seriation);
- use of pigmentation to discriminate individuals (linear discriminant analysis).

### Prevalence of pigmentation

#### Manual inspection for pigment

Five hundred and ten individual dolphins (Table 2) were used to assess the proportion of individuals with pigmentation patterns—of these, 95.3% exhibited pigmentation patterns.

**Table 2:** LDA leave-one-out cross-validation results when identifying the 169 dolphins.

	Features	% Correct	
		Top-1	Top-5
Unregistered	Contours	52.2	76.5
	Grid	70.5	82.5
	Contours + Grid	74.5	84.3
Registered	Contour	53.5	77.4
	Grid	69.2	82.6
	Contour + Grid	77.2	87.0

### Stability of pigmentation over time

#### Manual inspection for pigment change

**Figure 1:**
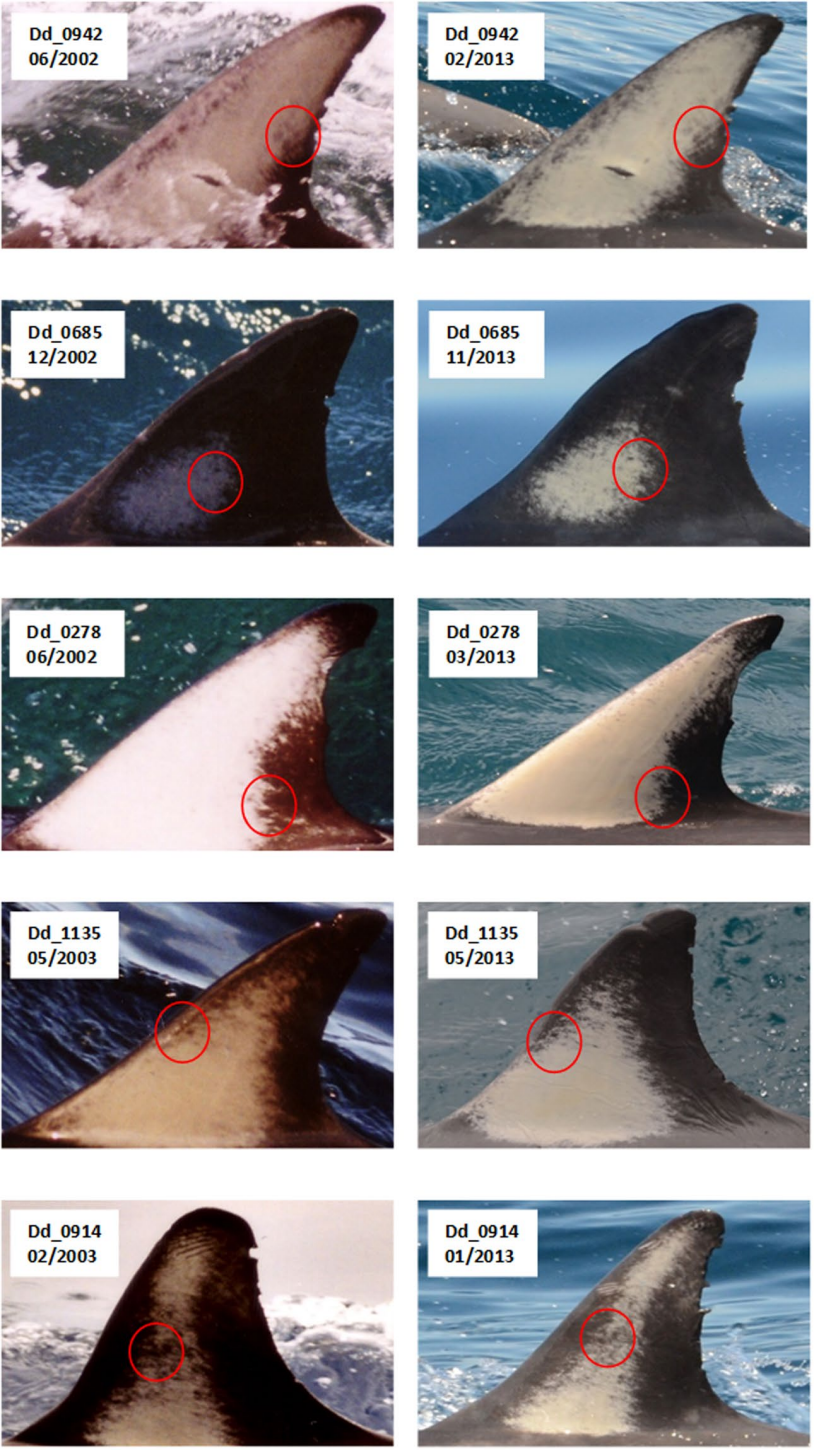
The first and last sighting of the five dolphins (from top: 942, 685, 278, 1135, 914) with the longest photographic epoch, suggesting stable pigmentation.

We manually (via visual inspection) examined the catalogue to see if there was evidence of pigmentation change over time. No substantial changes in pigmentation patterns were detected over time. An example of this is shown for five individuals with the longest photographic history (Fig. 1). None of the five individuals had substantial changes in pigmentation over a period of more than 10 years of available data.

#### Seriation test

**Figure 2:**
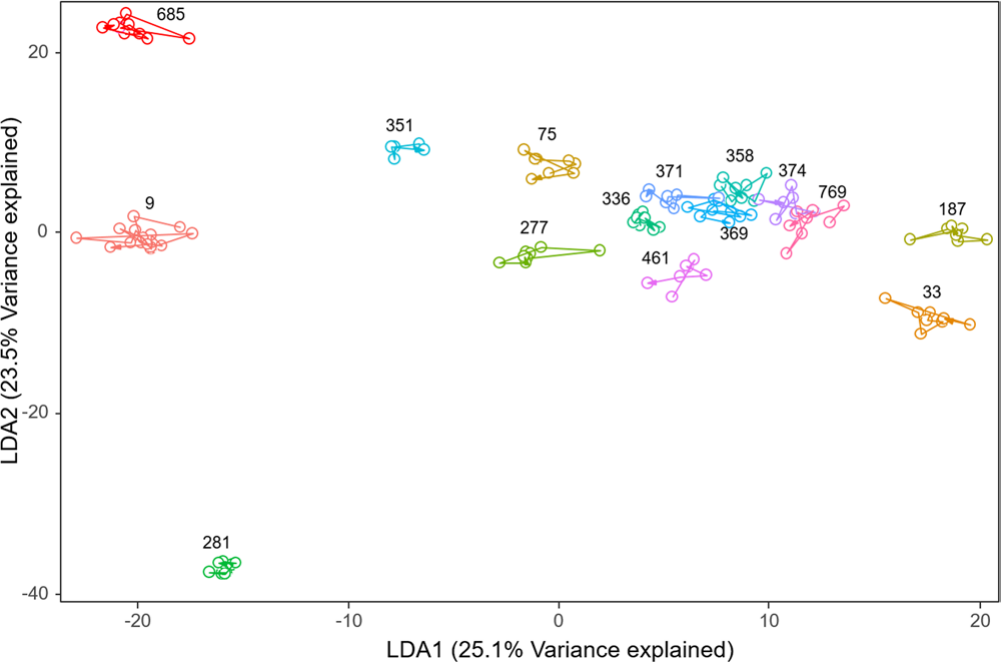
First two LDA axes discriminating between the 15 most-encountered (on separate occasions) dolphins (each identified by their number). Each circle represents an image from a dolphin and are connected by lines in the order of encounter date.

The seriation test used computer vision feature vectors derived from the 15 dolphins with the longest time series of images. The test examined the extent to which each dolphin’s pigmentation may have changed in a regular fashion. Visual examination of the feature vectors in linear discriminant analysis (LDA) space showed that images were positioned randomly around each individual’s centroid (Fig. 2). None of the 15 individual tests for seriation showed any evidence of any directional movements in the feature space (all seriation test $p$-values > 0.1 after Šidák correction). This suggests that the photographic order of fins (by date) is arbitrary, i.e. there is no evidence that the fin features are systematically changing over time (for example, pigmentation patches were not systematically growing or becoming darker or lighter over time).

### Discriminatory power of pigmentation

#### Manual matching

A reviewer unfamiliar with the catalogue manually matched 88% of images (*n = *614) as the correct individual based on pigmentation patterns alone. A reviewer familiar with the database manually matched 91% of images.

#### Linear Discriminant Analysis (LDA)

An LDA classifier was used to discriminate between individuals in the catalogue, using 142 computer vision features (i.e. contour and grid features) and assessed using leave-one-out cross validation (LOOCV). LDA was done on both registered (pose corrected) and unregistered dolphin images (Table 2). The classifier identified the correct dolphin on 77.2% of registered images. This is equivalent to a lift of 145, i.e. the classification result was 145 times better than randomly guessing the dolphin individual. For unregistered images, the accuracy dropped slightly to 74.5%. The LDA was also able to identify the correct dolphin in the top-5 results (ranked by the posterior probability) 87% and 84.3% of the time for registered and unregistered images, respectively.

## Discussion

For pigmentation patterns to be a useful feature in the context of identification they need to be: (i) prevalent within the population, (ii) stable over time and, (iii) have discriminatory power to detect individuals. We discuss these factors with respect to the common dolphin population within New Zealand (and cetaceans in general) below.

We found dorsal fin pigmentation patterns on common dolphins within the Hauraki Gulf to be prevalent among adults, i.e. visible on approximately 95% of photographed individuals. Traditional photo-id of common dolphins has relied on nicks/notches as the primary method of identification; however, less than half of adult individuals can be identified due to their low mark ratio^13^. This low mark ratio has likely discouraged photo-id studies on the genus globally, despite a distributional range spanning three oceans^19^. In contrast, pigmentation is nearly ubiquitous amongst adult common dolphins, suggesting that its use as the primary or even sole identification feature would dramatically increase the effectiveness of photo-id studies on *Delphinus*.

While pigmentation has been investigated over time using visual techniques for some poorly-marked delphinids^20,21^, no studies have statistically examined its temporal stability or discriminatory power for individual identification. In our study, neither visual nor statistical analyses found any evidence that the pigmentation patterns of adult common dolphins in the Hauraki Gulf changed over time. Photographs of some individuals over 11 years (between 2002 and 2013) showed a stable pigmentation pattern. Similar pigmentation longevity has been reported for other populations of cetaceans. For example, pigmentation patterns for northern bottlenose whales (*Hyperoodon ampullatus*) and blue whales (*Balaenoptera musculus*) in Canada have been found to be stable over a similar period^22,23^. One consideration is that pigmentation is known to change during maturation in some cetaceans. For example, beluga whales (*Delpinapterus leucas*) and Indo-Pacific humpback dolphin (*Sousa chinensis*) change from grey to white pigmentation with age^24^ and extreme changes in pigmentation also occur in the first year of post-natal development in humpback whales^25^. For common dolphins within the Hauraki Gulf, there is strong evidence of a difference in pigmentation patterns between age-classes, where immature animals have more uniform pigmentation and are less distinctive than adults^13^. However, since immatures are rarely marked with nicks and notches, we had no baseline identification to determine whether pigmentation could be used to uniquely identify immature individuals, so consequently this age-class was not considered in our analyses.

Pigmentation patterns clearly have discriminatory power when identifying individuals—manual observers could correctly identify 88–91 percent of individuals in the absence of nick or notch information. Moreover, after quantifying the pigmentation pattern, a machine learning method was capable of automatically discriminating common dolphins at levels similar to the manual observers. The LDA correctly identified 77% of individuals, and the correct individual was included in the top-5 model choices for 87% of the tested individuals. These automated identification results were approximately 145 times better than what would be expected if the pigmentation patterns had no discriminatory power.

Both classification methods (manual and automatic) assumed that the individual being classified is one of the existing catalogue entries. In practice, however, a consideration must be made as to whether the observation is an existing or a new individual. However, we considered this to be outside of the scope of this work and aim to investigate this in the future.

The use of automated identification should be further explored for delphinids. With the advent of digital imagery and powerful computers to analyse them, the use of technology and latest computer vision/machine learning algorithms can: 1) create a much more efficient workflow for biologists, and 2) capture and process much larger amounts of data than traditional methods allow. While manual matching of one individual to the catalogue takes approximately one hour, this was reduced to several minutes using automatic techniques. Automatic identification would increase the efficiency of matching large numbers of individuals by reducing both the amount of time spent cataloguing individuals and the number of errors introduced via the use of manual techniques. Unfortunately, robust automatic extraction of the fin from its environment is a challenging problem due to background water variation and specular lighting. Automation was, however, possible for the full projective transformation correction of the fin orientation using the ICP algorithm. The ICP algorithm proved to be useful for the registration of dorsal fins, even with some segmentation errors due to specular highlighting and/or water obstruction. However, our choice of features robust to different poses^26^ meant that registration gave limited improvements to the classification rate.

To maximise the efficacy of machine learning methods, we suggest that a paradigm shift is required in how ecologists typically create their catalogues. Traditional cataloguing involves manual matching of a single (‘best’) image of a ‘new’ individual against a single (‘best’) exemplar image of each catalogued individual. We suggest that ecologists should be storing and using as many photographs of each individual as possible for matching of new individuals to known catalogued animals. Keeping multiple photographs from each encounter and taking photographs using burst mode (or even high resolution video) will give far more computer-usable data on each individual, and therefore much more robust automatic identification will be possible. The use (and success) of state-of-the-art machine learning methods such as deep learning are heavily reliant on the amount of data collected for each individual^27^.

Some published studies have used dorsal fin pigmentation patterns to identify individual common dolphins worldwide^12,14–18^, but the prevalence, stability and discriminatory power of this feature has not been explored for these populations. The techniques and workflow presented here could be used to examine the viability of dorsal fin pigmentation as an identifying feature for other common dolphin populations. Moreover, a number of other delphinids exhibit pigmentation patterns, including Baiji (*Lipotes vexillifer*), Indo-Pacific humpback (*Sousa chinensis*), Irrawaddy (*Orcaella brevirostris*), pink river (*Inia geoffrensis*), tucuxi (*Sotalia fluviatilis*), Risso’s (*Grampus griseus*), and spotted (*Stenella frontalis*) dolphins^16,20,28–32^. Of these species, pigmentation prevalance is rarely investigated^29,32^. The suitability of pigmentation patterns on individuals of these species for computer-assisted photo-id can be investigated given the methodology described in this manuscript.

Future work aims to further improve the automated classification success rate by combining pigmentation features with dorsal fin nicks and notches, as well as collecting more data to apply advanced deep learning methods.

## Methods

### Ethics statement

The New Zealand Department of Conservation is the government agency responsible for the protection and management of New Zealand’s wildlife and the designation of special areas of conservation. No specific permission or permit was required for the fieldwork/data collection, as the Hauraki Gulf (36° 10′ to 37° 10′S, 174° 40 to 175° 30′E) is a public area. The current status of common dolphins is Least Concern (IUCN international status)^33^ and Not Threatened (New Zealand national status)^34^. The study did not involve the handling or manipulation of dolphins, but instead involved photo-id. As this method is non-invasive, no permissions or permits were required for data collection.

All research effort was performed in strict accordance with the New Zealand Department of Conservations recommendations for operating vessels around marine mammals, the Marine Mammals Protection Act 1978 and Marine Mammals Protection Regulations 1992.

To minimise disturbance to animals, vessels were restricted to moving towards animals at a slow speed (~5.0 kts), travelling on a parallel course and approaching from the rear. The behaviour of dolphins around survey vessels were constantly monitored. If strong behavioural responses were observed (e.g., continued loud exhalations or tail slaps), the survey protocol was suspended and the encounter was terminated.

### Field methods

**Figure 3:**
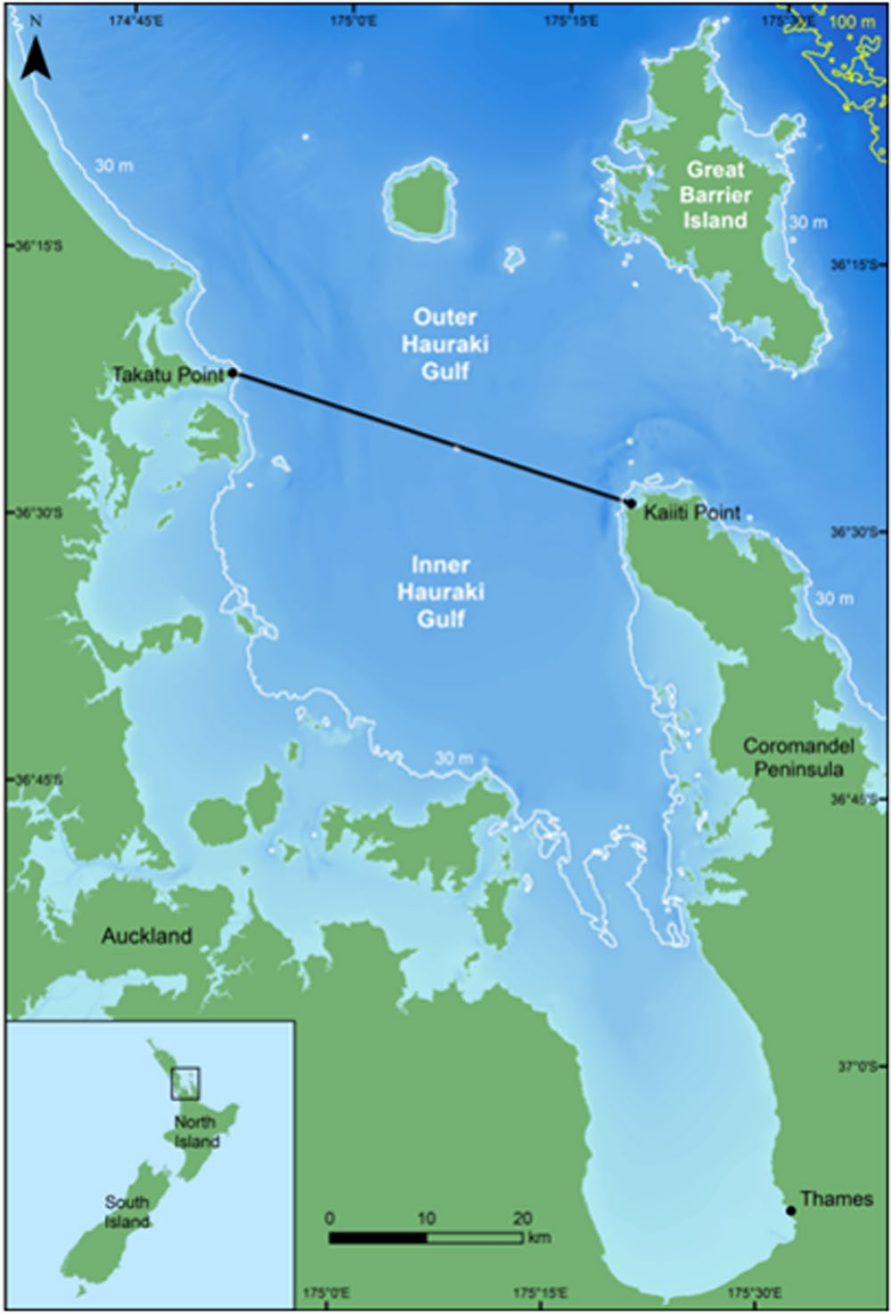
Study area, the inner Hauraki Gulf (HG), New Zealand. The solid black line (from Takatu Point to Kaiiti Point) indicates the boundary between the inner and outer HG. The white and yellow lines indicate the 30 m and 100 m isobaths, respectively. Bathymetry is indicated by darker shades of blue which represent deeper waters (Source: NIWA; Mackay *et al*. 2012). Inset shows the location of the HG and North Island, relative to New Zealand.

A total of 522 days of opportunistic photo-id surveys were conducted between February 2002 and December 2009 in the inner Hauraki Gulf (Latitude 36° 10′ to 37° 10′S, Longitude 174° 40′ to 175° 30′E; Fig. 3) on the north-eastern coastline of the North Island, New Zealand. In addition, 419 days of dedicated photo-id surveys were conducted in the same region between January 2010 and December 2013. Images were collected from three vessels: two 5.5 m research vessels and a 20 m commercial catamaran. Surveys on the research vessels were conducted in good visibility (≥1 km), swell <1 m, and Beaufort Sea State (BSS) ≤3 ( ≤BSS 4 when onboard the commercial platform)^35^. Two to five trained observers conducted concurrent photo-id sessions, following standardised methods^36^. Photographs were taken using Nikon D90 and D7000 SLR cameras, equipped with Nikon 100–300 and 100–400 mm lenses, respectively. Images were taken approximately perpendicular to the dolphin heading, and within 25 m^9,37^. Only one side of the dorsal fin (left) was photographed for consistency purposes. Putative age classes of dolphins were classified as either immature (including neonates, calves and juveniles) or adult by estimating the size and independence from the putative mother for each individual^13,37^. Immature common dolphins were unmarked and were therefore excluded from all analyses.

### Photographic-quality grading

Taking high quality photographs of common dolphins is difficult because the photographer typically has only a fraction of a second to capture the image with little idea of exactly where the animals will surface. Images of the same dolphin dorsal fin taken at different times vary due to a variety of reasons, including:

1. Differences in lighting conditions;
2. Occlusion due to environment (waves, rain, sea spray or water sheeting over the skin);
3. Apparent changes in dorsal fin shape due to variation in the orientation of the animal relative to the camera’s image plane (perspective distortion). Ideally, the camera’s viewpoint should be perpendicular to the dorsal fin plane because this gives the best view of the fin and keeps the pose consistent between photographs; however, this is almost impossible in practice.

Because of such a wide range in possible image quality of field photographs, we applied strict grading protocols, for example, based on photographic-quality (PQ) criteria with the aim of minimising bias and reducing misclassification. Images were graded according to PQ criteria. Each image was assigned a value based on the following categories: clarity and focus (scored as poor - 1, reasonable -4, or excellent - 9); degree of contrast (scored as 1, 3, or 9); orientation/angle (scored as poor - 1, reasonable − 2, or excellent -9), and dorsal fin edge visibility (scored as poor/reasonable - 1 or excellent - 8) (adapted from Urian *et al*. and Nicholson *et al*.^38,39^; Supplementary Table S1). Scores for each category were weighted so that inadequate quality in one category alone would ensure an image was rated as poor^39^. Values for each category were then summed to produce an overall image quality score, from poor to excellent (Supplementary Table S2). Only images that were rated as ‘good’ or ‘excellent’ were retained within the photo-id catalogue^13^.

### Photo-id catalogue

**Figure 4:**
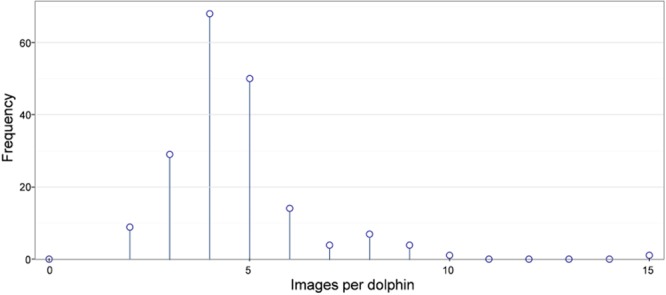
The number of images per individual in the photo-id database (mean = 4.6).

A photo-id catalogue was developed to identify adult common dolphins. The catalogue contained 2,399 individuals with nicks/notches that were used to identify animals with high certainty. Pigmentation patterns were also used as a secondary identifier when present. The catalogue was collated manually and each new prospective dolphin was carefully examined. All matches were scrutinised by at least two independent experienced observers before being assigned a unique identification code^13^. For the purposes of this study, a subset of individuals in the catalogue was selected based on the following criteria: at least two sightings if one or more of these were from the opportunistic surveys (2002 to 2009), otherwise at least four sightings. We used a lower sightings threshold for opportunistic surveys to include more individuals with a long period between resighting (Fig. 4). Using these criteria resulted in a subset of 779 images of dorsal fins from 169 unique adult common dolphins (hereafter referred to as *the catalogue*).

### Quantifying pigmentation in images of dolphin dorsal fins

This section outlines the process that was used to quantify pigmentation patterns in order to perform the statistical analysis described in the next section.

#### Pre-processing

Each image was cropped to show the dorsal fin of a single dolphin, which was then manually segmented from the background using Adobe Photoshop CS5^40^. After the background removal, images were converted from colour to grey-scale. Furthermore, to account for the differences in camera exposure settings and global illumination changes, the pixel values were normalised by subtracting the mean and dividing by the standard deviation of all pixels that fell within the fin area. This and all further image processing was performed in MATLAB^41^.

#### Feature extraction

To quantify the pigmentation patterns, we extracted measurable properties that were representative of the patterns. In the pattern recognition field, these properties are called ‘features’ and the process of computing them from the raw image pixel values is called ‘feature extraction’. In this context, a feature is a numeric value, obtained in a way described below, and is synonymous to an explanatory variable in regression analysis^42^.

#### Robust subdivisions of the fin

We standardised the rotational position of each fin using the fin base—this was defined as a line passing through the point where the leading edge of the fin flows into the dolphin’s body (forming an inflection point in the outline) and running parallel to the body direction. Following Gilman *et al*.^43^, we corrected for perspective distortion (i.e. changes in dorsal fin shape due to variation in fin orientation relative to the camera’s image plane) by registering fin contours using the iterative closest point algorithm.

We attempted to find a subdivision scheme that would give the best trade-off between discriminatory power and robustness to perspective distortion. We explored a number of different ideas and settled on two subdivision schemes: (1) a *contour-based* subdivision based on the fin outline; (2) a *grid-based* subdivision based on the position of the base of the fin. We hypothesised that subdividing the fin and summarising pixel values within each division with a robust statistic would result in features that were relatively invariant to changes in fin orientation (relative to the camera), blurring, poor resolution, occlusions, specular highlights and reflection artefacts, all of which are inherent to photography in the field.

#### Grid features

**Figure 5:**
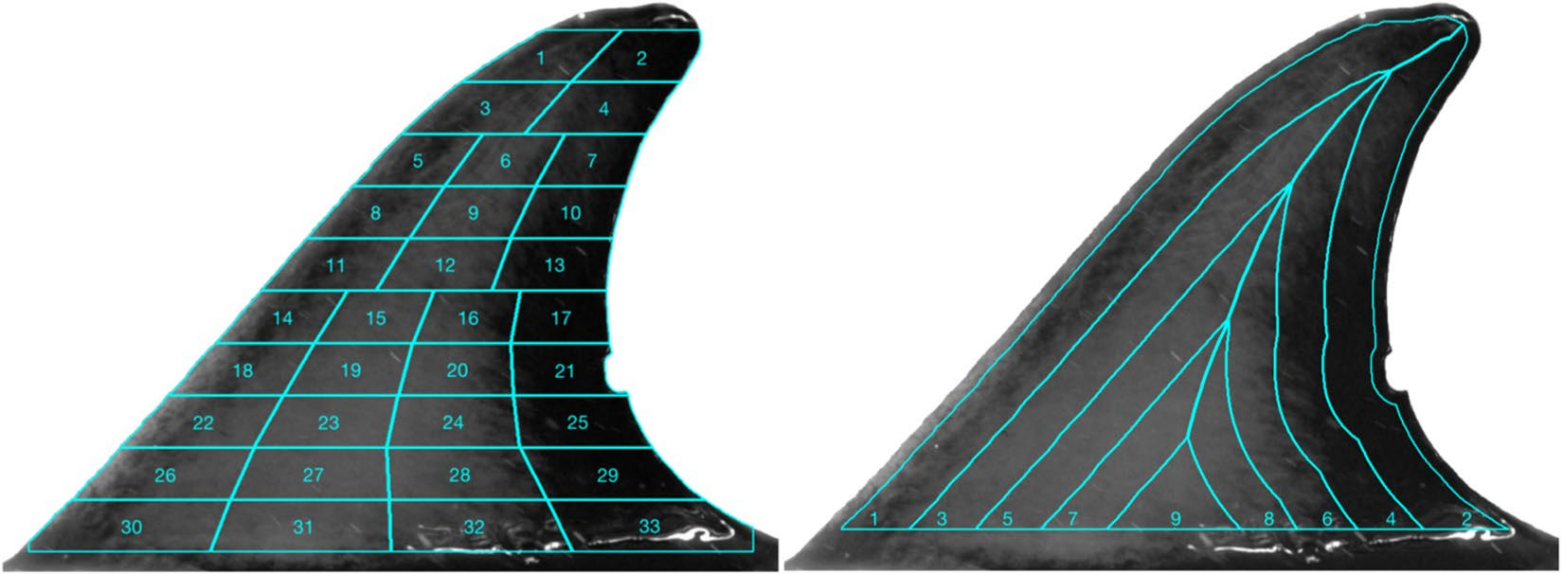
Feature creation from a dorsal fin image, using **(A)** Grid-based subdivisions, and **(B)** contour-based subdivisions.

As the base of the dorsal fin can be identified in a relatively robust manner, the fin was divided into 10 equal subdivisions along an axis perpendicular to the base, with the upper limit of the grid area defined as half the segment’s height from the top of fin (we ignored top-most part of the fin as it often contained specular highlights). The bottom five subdivisions were further split into four patches, the three central horizontal subdivisions were split into three patches, and the top two horizontal segments were split in half (see Fig. 5), resulting in 33 image patches arranged in a grid-like fashion.

#### Contour features based on the closest distance from the fin edge

A distance transform was computed on the outline of the fin—each pixel that fell within the fin area was allocated a value corresponding to the Euclidean distance to the nearest point on the fin outline. Equidistant iso-contours were computed and used to subdivide the fin area into five equal-width segments running along the fin outline and located progressively further towards the centre (Fig. 5). These divisions were made, as we observed the area between these iso-contours to be robust to moderate changes in dorsal fin perspective. To give these features more discriminating power each area was subdivided further into two parts in the following way. The fin’s medial axis was computed by tracking the ridge of the distance transform (essentially a set of points equidistant from fin’s leading and trailing edges). This medial axis was used to further subdivide four of the five segments (except for the smallest centre-most segment) into left and right halves, resulting in nine contour-based image patches.

#### Summary Statistics

Within each of the image patches falling under the 42 subdivisions (i.e. 33 grid-based patches + 9 contour-based patches), the following summary statistics were calculated on the distribution of normalised greyscale pixel intensities:

1. Mean,
2. Median and
3. Interquartile range (IQR).

In addition, 16 inter-divisional features were calculated (Table 3), giving a total of 142 features that characterised each image.

**Table 3:** A list of interpatch features, where *std(gridMeans)* denotes standard deviation of the distribution of the 33 grid segment means, *std(contMeans)* denotes standard deviation of the distribution of the 9 contour segment means etc.

Interpatch Means	Interpatch Median	Interpatch IQR
std(gridMeans)	std(gridMedians)	std(gridIQRs)
IQR(gridMeans)	IQR(gridMedians)	IQR(gridIQRs)
		mean(gridIQR)
		median(gridIQR)
std(contMeans)	std(contMedians)	std(contIQRs)
IQR(contMean)	IQR(contMedians)	IQR(contIQRs)
		mean(contIQRs)
		median(contIQRs)

**Figure 6:**
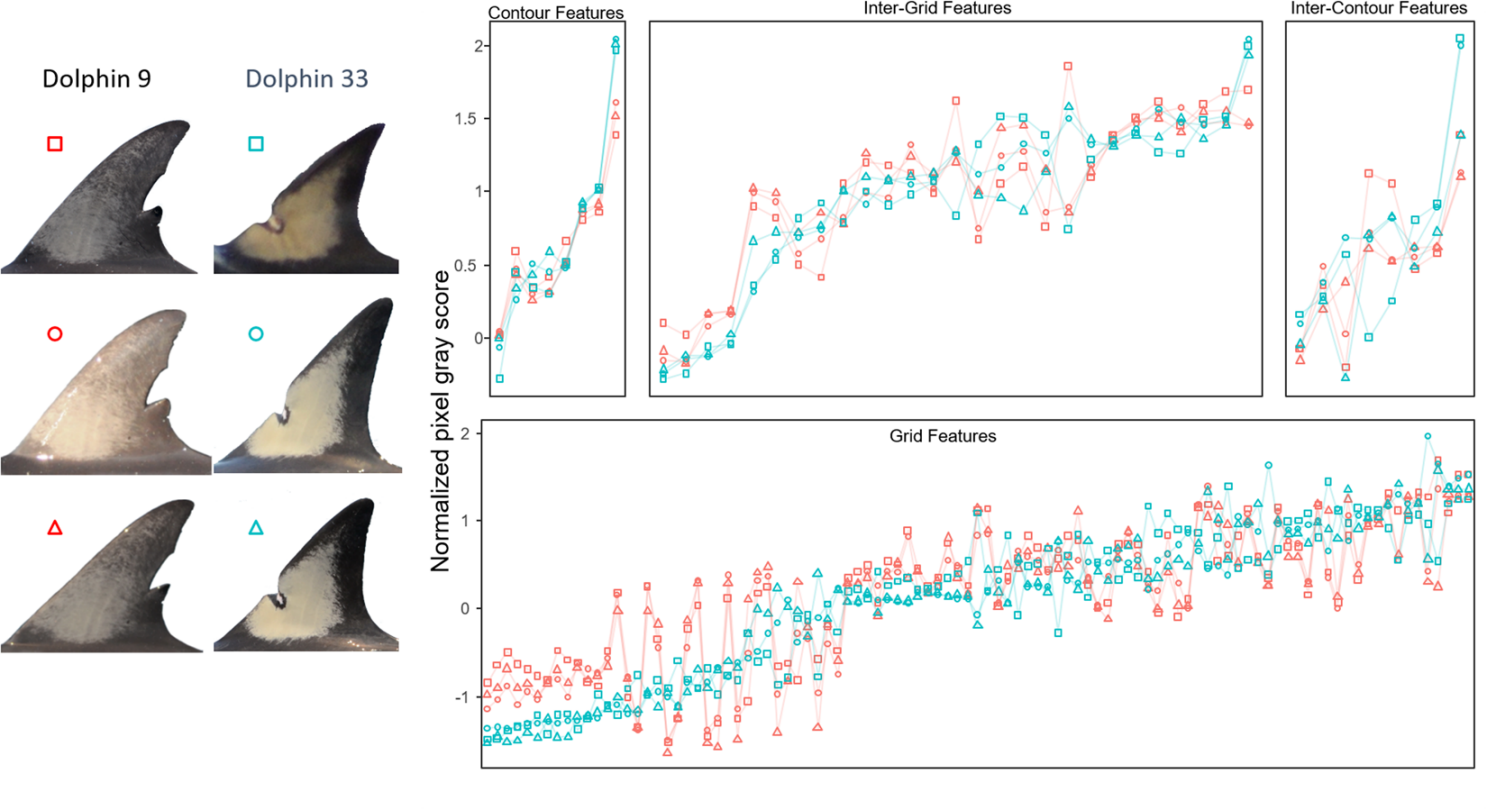
Line plot presenting multivariate feature vectors of six different photos (two individuals each with three images) suggesting a pigmentation ‘signature’. Features were grouped into six classes and ordered by feature group value of dolphin 33.

The values of the 142 features, composed into 142-dimensional vectors, form a multidimensional ‘feature space’ that was used to discriminate between photographs from different dolphins. Figure 6 shows six exemplar feature vectors, three derived from photographs of dolphin 9 and another three derived from photographs of dolphin 33. It can be observed that images from the same dolphin result in relatively similar feature vectors, but different dolphins tend to sit in somewhat different areas within the high dimensional feature space.

### Datasets

Three datasets were used to examine the prevalence, stability and discriminatory power of pigmentation (Table 4), and the analysis techniques are described below:

**Table 4:** Summary of the objective, method and data set used for each visual or statistical analysis completed.

Objective	Method	Dataset used	Visual or statistical analysis
Prevalence of pigmentation	Manual inspection for pigment	Dataset of 1,680 dorsal fin images from 510 unique adults collected over 31 photo-id sessions (days). This dataset only included images of adult individuals of good- or excellent photo-quality, but were not included into the catalogue. Photo-id sessions included in this database (n = 31) were chosen by random sub-sampling of the total photo-id sessions (n = 941). This dataset was used because it was not biased by cataloguing purposes, i.e. the catalogue images were deemed to be more likely to have pigmentation since its presence was used as a secondary identifier.	Visual
Stability of pigmentation over time	Manual inspection for pigment change	The catalogue - dataset of 779 dorsal fin images from 169 unique adults.	Visual
	Seriation test	Dataset of 129 dorsal fin images from 15 unique adults. These 15 individuals were the most encountered dolphins within the catalogue.	Statistical
Discriminatory power of pigmentation	Manual matching	The catalogue - dataset of 779 dorsal fin images from 169 unique adults.	Visual
	Linear Discriminant Analysis		Statistical

### Prevalence of pigmentation

#### Manual inspection for pigment change

For each photo-id session, individuals were sorted into those showing pigment (i.e. any image with light or dark pattern, regardless of the pattern’s shape or size) versus those with no pigment.

### Stability of pigmentation over time

#### Manual inspection for pigment change

We manually (via visual inspection) examined the catalogue to see if there was evidence of pigmentation change over time. Particular attention was paid to all photos of the five common dolphins with a photographic history spanning more than 10 years.

#### Seriation test

We selected the top 15 dolphins which had a) the longest time series of images and b) the most images taken each photo-id session, to examine the extracted feature vectors for seriation (i.e. the extent to which each dolphin’s pigmentation may have changed in a regular fashion). If the seriation test null hypothesis is true, then the photographic order of fins (by date) is arbitrary and we can infer that the features of our fin are not systematically changing over time. Given that there were 15 separate tests, we used the Šidák correction to give a family-wise error rate of 5%^44^, i.e. the null hypothesis was rejected if the p-value was less than ${\alpha }_{{SID}}\,=\,0.003414$. We also visualised changes over time using the axes from a Linear Discriminant Analysis (LDA) (performed using the R package ‘MASS’^45^), discriminating between the 15 dolphins. Each image was a separate point within the LDA ordination, and images were joined using the date of the photographs (from earliest to more recent image).

### Discriminatory power of pigmentation

#### Manual matching

**Figure 7:**
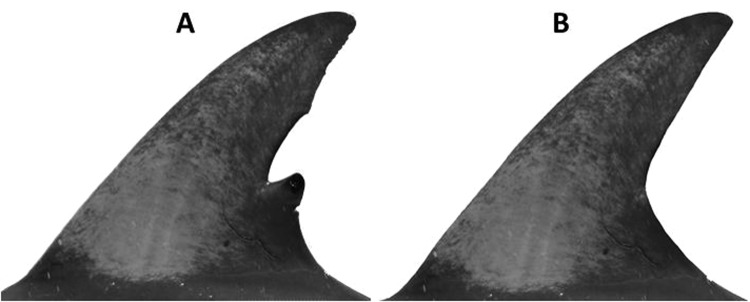
Nicks/notches were removed (dolphin 9 shown) when manually assessing the efficacy of pigmentation as a sole identifier: **(A)** the original image; **(B)** the modified image with the external boundary smoothed.

To minimise bias, we removed any visible nicks/notches from all images within the catalogue (779 images). The external boundary of the leading and trailing edges of each dorsal fin was ‘smoothed’ by filling in any nicks/notches with realistic skin texture using Adobe Photoshop CS5 (Fig. 7). The background was also removed, with the aim that pigmentation patterns were the only remaining information that could be used to match individuals. One random image from each dolphin was withheld to create a catalogue of individuals (n = 169). A reviewer (unfamiliar with the dataset) then attempted to match the remaining images (n = 614) to this catalogue and calculated how many images were correctly classified. A second reviewer (familiar with the dataset) also replicated the experiment.

#### Linear discriminant analysis

To determine if we could classify individuals using just their pigmentation pattern, we used a regularised LDA, and estimated its efficacy using leave-one-out cross validation. We regularised the LDA due to the high dimensionality of the feature space and relatively small number of images per dolphin. Regularisation was done using a James-Stein shrinkage estimate of the covariance matrix implemented in the R package sda^46,47^; this reduces the off-diagonal elements of the covariance matrix towards zero in an effort to obtain a biased but less variable estimator.

## Electronic supplementary material

Supplementary Tables

## Data Availability

The datasets generated during and/or analysed during the current study are available from the corresponding author on reasonable request.
