# Supplementary material for: Examining the viability of dorsal fin pigmentation for individual identification of poorly-marked delphinids
Source: Sci Rep. 2018 Aug 22;8:12593. doi: 10.1038/s41598-018-30842-7 (PMC6105684; doi:10.1038/s41598-018-30842-7)
Supplement: Supplementary file 1 — Supplementary Tables [file 41598_2018_30842_MOESM1_ESM.docx]

Title: Examining the viability of dorsal fin pigmentation for individual identification of poorly-marked delphinids

**MDM Pawley^1,*,+^, KE Hupman^1,2,+^, KA Stockin^1^, and A Gilman^1^**

**^1^Institute of Natural and Mathematical Sciences, Massey University, Auckland, 0745, New Zealand**

**^2^ National Institute of Water and Atmospheric Research, 301 Evans Bay Parade, Wellington, 6021, New Zealand**

*****[M.pawley@massey.ac.nz](mailto:M.pawley@massey.ac.nz)

**^+^**these authors contributed equally to this work

**Supplementary Table S1**

Description of attribute criteria used to examine the photographic quality (PQ) of common dolphin images in the Hauraki Gulf, New Zealand. Images were assessed according to focus, exposure, orientation, and visible percentage (adapted from [1-2]). When assessing quality criteria each attribute was considered independently to avoid bias/contradictions between categories being assessed.

| *Attribute* | Description | Score |
| --- | --- | --- |
| *Focus* | - Poor: considerable blur - general outline and/or details are not visible - Reasonable: some blur - general outline visible and small nicks may not be entirely visible - Excellent: no blur - sharp outline and all details are visible | 9  4  1 |
| *Photographic exposure* | - Poor: Under- or over-exposed, only some details are seen - Reasonable: A little light or dark but all details are clearly seen - Excellent: No over or under exposure and all details and outline are visible | 9  3  1 |
| *Orientation (angle between camera axis and fin plane)* | - Poor: ≤45° to Perpendicular - Reasonable: >45° - Excellent: 90° | 9  2  1 |
| *Percentage visible* | - Poor/reasonable: The leading and trailing edges of the dorsal fin are partially obscured - Excellent: The leading and trailing edges of the dorsal fin are fully visible | 8  1 |

**Supplementary Table S2**

Photographic quality (PQ) categories used to examine adult common dolphin images in the Hauraki Gulf, New Zealand. Images were classified as: a) poor; b) fair; c) good, or; d) excellent quality.

| **PQ category** | **Score** | **Example** |
| --- | --- | --- |
| *Poor* | ≥11 | 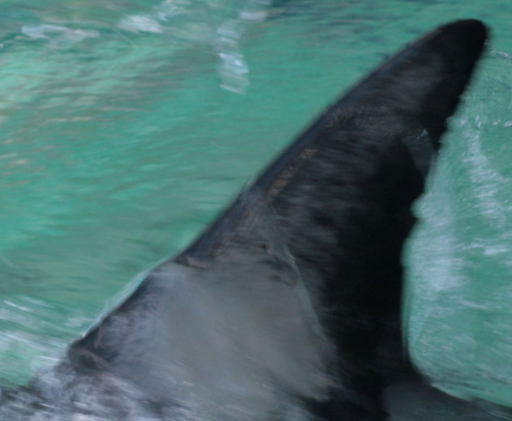 |
| *Fair* | 10 | 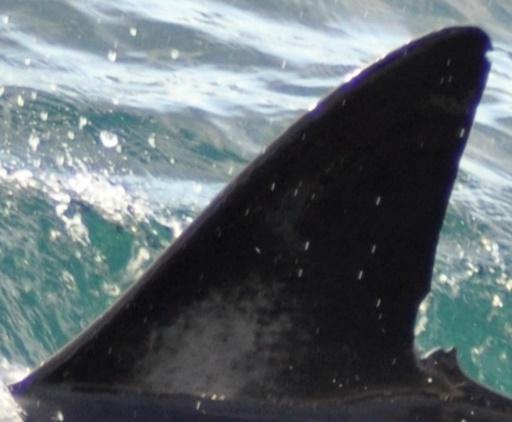 |
| *Good* | 7–9 | 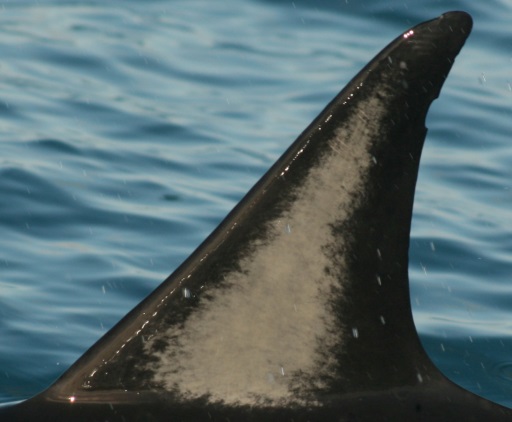 |
| *Excellent* | 4–6 | 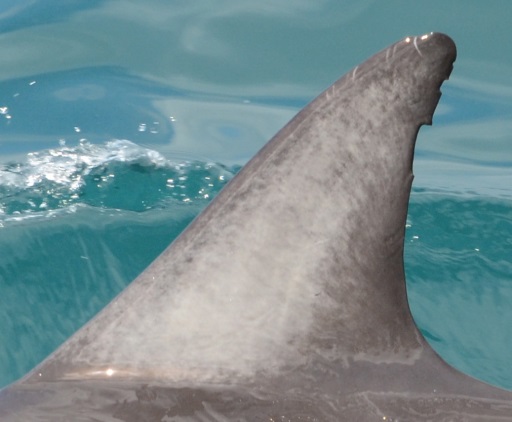 |
